# Identification of secondary metabolites containing a diketopiperazine core in extracts from myxobacterial strains with growth inhibition activity against a range of prey species

**DOI:** 10.1099/acmi.0.000629.v4

**Published:** 2023-10-05

**Authors:** Emily J. Radford, David E. Whitworth, Gordon Allison

**Affiliations:** ^1^​ Department of Life Sciences, Aberystwyth University, Aberystwyth, SY23 3DD, UK

**Keywords:** antimicrobial, diketopiperazine, GC-MS, myxobacteria, predation, secondary metabolites

## Abstract

Myxobacteria produce a variety of bioactive secondary metabolites, and with a wealth of under-researched species they hold vast potential for undiscovered compounds. With the ever-increasing need for new antibiotics, the development of novel therapeutics is vitally important. Therefore, this study aimed to extract and elucidate antimicrobial metabolites from the following myxobacteria: *Myxococcus xanthus* CA010 and AB022; *Corallococcus exiguus* DSM 14696^T^; *Myxococcus stipitatus* DSM 14675^T^; and *Corallococcus aberystwythensis* AB050A^T^. Metabolite mixtures were extracted in acetone from XAD-16 resin incubated in liquid cultures and analysed using GC-MS. Bioactivity was identified using a growth inhibition assay against a panel of clinically relevant prey species including Gram-positive and Gram-negative bacteria and a fungus. Growth of *Klebsiella pneumoniae* and *Enterococcus faecalis* was most affected by the metabolite mixtures and the mixtures from AB022 and AB050A^T^ were effective against the most prey. GC-MS analysis revealed metabolites with roles in the synthesis and degradation of amino acids and fatty acids, but also identified compounds A and B with a diketopiperazine (DKP) core. With previously confirmed bioactivity of compound A, it is suggested that these DKP compounds are contributing to the antimicrobial activity observed. Furthermore, many compounds could not be identified and so these unknowns present further potential for novel bioactive compounds.

## Data Summary

The authors confirm all supporting data, code and protocols have been provided within the article or through supplementary data files, available in the online version of this article. In addition, the raw GC-MS data have been uploaded to a public access data repository with the DOI: 10.17605/OSF.IO/8BZKT.

## Introduction

Myxobacteria (members of the phylum *Myxococcota *) produce an abundance of secondary metabolites with a range of bioactivities. This trait is driving interest within the scientific community for their potential as producers of novel compounds, as these metabolites represent the possibility for alternative therapeutics which could dramatically impact the human, animal and plant health sectors. Therefore, continued investigation in this area is vital.

In the soil microbiome community, there is a vast spectrum of bacterial interactions and competition for resources. Myxobacteria obtain growth substrates through predator/prey interactions with both Gram-positive and Gram-negative bacteria and fungi, but specific species show predation preferences for specific prey. However, the phylogenetic relationship between myxobacterial isolates does not accurately predict their predatory activity, suggesting the importance of horizontal gene transfer in the acquisition of predation factors which dictate their prey range [1]. Furthermore, there seem to be few myxobacterial genes that confer widespread predatory activity, and instead myxobacterial genomes possess many prey-specific predation genes, which explains the wide range of predatory activity that does not correlate with prey phylogeny [2].

Within any taxonomic level a pan-genome can be defined which is the entire set of all genes present from all members of that clade. Subsequently, a core genome and accessory genome can be defined, meaning the genes present in all members of the clade, and genes present in at least one member (but not all), respectively. From a sample of ten strains of the species *Corallococcus exiguus* around 25 % of an individual strain’s genome but over half of the pan-genome comprised accessory genes, highlighting the variation between individual isolates even of the same species [3]. The accumulation of many predatory genes in the accessory genome presumably confers a substantial advantage as it can allow efficient predation should a particular prey be encountered in the future, even though that gene may not be an immediate advantage. This is an unusual evolutionary strategy as the replication of a typical myxobacterial genome which is large with high %GC (which is more metabolically expensive to produce than a high %AT genome) is linked to a slower growth cycle [4].

A key predatory activity of myxobacteria is the production of biologically active secondary metabolites. Myxobacterial isolates have diverse profiles of biosynthetic gene clusters (BGCs) which produce secondary metabolites, and which are linked to their high genetic diversity. The myxovirescin family of antibiotics was first identified as being produced by *Myxococcus virescens* with myxovirescin A being the major constituent and having most significant activity against prey [5]. Myxovirescin is also produced by other members of the genus *Myxococcus* including *Myxococcus xanthus *. It has been demonstrated that myxovirescin is a selective antibiotic factor as an *M. xanthus* mutant lacking the ability to produce myxovirescin could not produce a zone of inhibition on an *Escherichia coli* lawn but did inhibit *Micrococcus luteus* growth – despite the fact that the original non-mutant *M. xanthus* strain showed predation against both prey species [6]. Furthermore, under starvation conditions, there was no difference in *Escherichia coli* killing in the presence or absence of myxovirescin, suggesting this antibiotic plays an important role against metabolically active cells but is not a key predation factor against metabolically quiescent cells [6]. Other myxobacterial bioactive metabolites include the antibacterial compound corallopyronin A (from the species *Corallococcus coralloides *), and the antifungal metabolites ambruticin and haliangicin (isolated from *Polyangium cellulosum* var. *fulvum* and *Haliangium luteum* respectively) [7–9]. Extraction and synthesis of the halogenated macrolide containing compound chlorotonil A has revealed significant activity against all life stages of the malaria-causing protozoan *Plasmodium falciparum* and shows activity through a different mode of action to traditional malaria treatments, suggesting this compound has the potential to improve malaria treatment [10–12]. In addition, nannocystin A (isolated from a *Nannocystis* sp.) demonstrated significant antiproliferative activity with IC_50_ values of 1.0–12 nM against a variety of cell lines, highlighting it as a lead compound for anticancer treatments [13]. The examples mentioned here are just a small sample of the discoveries made and represent a group of organisms which may have further untapped potential for a vast range of activities and applications. Therefore, biologically active metabolites isolated from myxobacteria represent a vital source of novel therapeutics that could greatly impact the human, veterinary and plant health sectors.

It has been found that myxobacterial predators with substantial activity against a single prey species tend to also display significant predatory activity against multiple prey species, making these predators particularly important [1]. Furthermore, secondary metabolite production represents a key component of myxobacterial predation, and therefore we investigated the potential antimicrobial metabolites produced by predatory myxobacterial species. To accomplish this, metabolites were extracted from myxobacterial cultures, antimicrobial activity was assayed against a panel of clinically relevant prey organisms, and metabolites were characterized using structural elucidation techniques such as GC-MS. It was found that the metabolite mixtures from all five myxobacterial strains had a unique profile of activity against the prey panel in a liquid growth inhibition assay. GC-MS analysis revealed a variety of metabolites including some with a diketopiperazine core that are suggested to contribute to the antimicrobial activity observed. Many other metabolites could not be identified from library matches, and so there is potential for novel bioactive compounds to be found among these. This investigation presents myxobacterial metabolites with the potential for development as novel therapeutics and highlights the presence of further compounds which could not be identified using the methods employed in this investigation.

## Methods

### Bacterial culture and preparation

Five myxobacterial strains were used in this study (Table 1). These were maintained on DCY agar plates (2 % casitone, 0.2 % yeast extract, 10 mM Tris, 8 mM MgSO_4_, 1.5 % agar, pH 7.8) at 30 °C. The leading edge of the swarming colony was inoculated into 100 ml liquid DCY media (as before, but omitting the agar) and incubated in a shaking incubator at 30 °C and 180 r.p.m. for 5 days. Cultures going forward for metabolite extraction were incubated in DCY liquid media as above with the addition of 2 % Amberlite XAD-16 resin beads (Sigma). This was repeated in triplicate.

**Table 1.** Species and strains of prey and myxobacterial predators used in this study

	Species	Strain	Gram stain	Genome accession	Contigs	Predatory rank*	Reference
Prey	*Candida albicans*	NCTC 32	na				
	* Escherichia coli *	TOP10	Negative				
	* Klebsiella pneumoniae *	ATCC 700603	Negative				
	* Enterococcus faecalis *	ATCC 29212	Positive				
	* Staphylococcus aureus *	ATCC 29213	Positive				
	* Staphylococcus saprophyticus *	Wild-type laboratory strain	Positive				
Myxobacterial predators	* Corallococcus aberystwythensis *	AB050A^T^	Negative	SAMN10026036	625	107	[17]
	* Corallococcus exiguus *	DSM 14696^T^	Negative	PRJNA547735	36	na	[17]
	* Myxococcus stipitatus *	DSM 14675^T^	Negative	CP004025	1	na	[32]
	* Myxococcus xanthus *	AB022	Negative	VHLD01000001	257	105	[2]
	* Myxococcus xanthus *	CA010	Negative	VHLA01000001	250	1	[2]

*Predation data (diameter of the zone of killing after 4 days) from Livingstone *et al*. [1] across ten prey species were averaged to give a mean for predation activity for each of the 114 strains. These means were ranked with 1 being the best and 114 being the worst predator on average. This is represented as ‘predatory rank’.

Prey species (see Table 1) were maintained on LB agar plates (1 % NaCl, 1 % tryptone, 0.5 % yeast extract, 1.5 % agar) and incubated at 30 °C before being stored at 4 °C. Single colonies were inoculated into 100 ml liquid LB media (as before, omitting the agar) and incubated in a shaking incubator at 30 °C and 180 r.p.m. for 24 h to obtain a dense culture.

### Phylogenetic analysis

The relatedness of the myxobacterial and bacterial prey strains was assessed through the construction of a phylogenetic tree created from the 16S rRNA gene sequences using the ‘one-click’ function on the online web-service Phylogeny.fr [14]. muscle and Gblocks facilitated multiple sequence alignment, and PhyML alongside TreeDyn produced the maximum-likelihood tree using default settings. The type strain of each of the prey species was identified from the List of Prokaryotic names with Standing in Nomenclature (LPSN) [15] and the 16S sequences were accessed from the NCBI database and downloaded in FASTA format. A multiFASTA file was compiled for submission to Phylogeny.fr.

### Biosynthetic gene cluster prediction

A prediction of the BGCs and their associated metabolites was provided using antiSMASH version 6.1.1 (accessed via the online user interface available at https://antismash.secondarymetabolites.org/#!/start) [16] into which the whole genome sequence was uploaded. Each sequence was accessed through the NCBI online database and downloaded in GBFF format before being submitted for analysis with antiSMASH using all available features with a relaxed detection strictness.

### Metabolite extraction

After 5 days of growth, triplicate myxobacterial cultures containing XAD-16 resin were subjected to centrifugation at 5000 ***g*** for 10 min and the supernatant was removed. The beads were then filtered and washed twice using sterile water, sonicated using a Misonix 3000 sonicator (total run time of 2 min in 10 s bursts with 10 s cooling between at a power of ~27 W) to lyse any remaining cells before being washed a third time and spread out on filter paper to dry. Once dry, 4 ml of acetone was added before centrifugation at 5000 ***g*** for 10 min. The supernatant was removed and dried under a N_2_ stream. The residue was further freeze-dried overnight before being resuspended in DMSO at a concentration of 0.4 g ml^−1^.

### Growth inhibition assay

A 96-well microplate was prepared with outer cells containing only buffer to avoid position effects, and triplicates of each control and test compound positioned sequentially across the plate. Each well had a total volume of 250 µl, with 2.5 µl of prey cells resuspended in TPM (10 mM Tris, 1 mM KH_2_PO4, 8 mM MgSO_4_) in each well (excepting media only control wells), 2.5 µl control/test compound in each well (except prey-only standard wells), and liquid LB media making up the remainder. A 1 : 1 mixture of lysozyme (10 mg ml^−1^) and SDS (0.5%) was used as a positive control, while a 10^−1^ dilution of DMSO was used as a negative control. The prepared plate was incubated under gentle orbital shaking conditions (120 r.p.m.) at room temperature for 24 1 h cycles in a HIDEX sense 96-well microplate reader where the optical density (OD_600 nm_) was measured every hour. The data were recovered at the end of the 24 cycles and compiled into a spreadsheet for analysis.

### Metabolite characterization and structural elucidation

A 10 µl aliquot of the metabolite mixture in DSMO (diluted to 0.2 g ml^−1^ to ensure sufficient volume) from each strain was dried thoroughly under an N_2_ stream. Each sample was subjected to methoximation–trimethylsilyl derivatization. This involved the addition of 30 µl of methoxamine/pyridine mixture (20 mg ml^−1^), before capping and incubating in a 90 °C heat block for 15 min. Following this, 20 µl of BSTFA [*N*,*O*-*bis*(trimethylsilyl)trifluoroacetamide] was added and the incubation repeated. The resulting sample was then analysed by GC-MS. GC-MS analysis was performed on a Shimadzu GCMS-QP2010 Ultra coupled with an AOC-20s autosampler and an AOC-20i autoinjector. The injection temperature was 280 °C and at the point of injection the column oven was held at 80 °C. This flow of carrier gas was held at a constant linear velocity of 36 cm s^−1^ and samples were injected using a split ratio of 25 : 1 onto a HP5 column. The injection temperature was held for 2 min and then increased to 300 °C at a rate of 15 °C min^−1^, held for 2 min and then finally raised to 330 °C at a rate of 50 °C min^−1^ and held for 5 min to purge the column. The temperatures of the transfer line, the ion source and the quadrupole were 330, 230 and 150 °C respectively. Mass spectra were monitored by quadrupole scanning over a range of 50 *m*/*z* to 550 *m*/*z*. Tuning and all other settings of the mass spectrometer were according to the manufacturer’s recommendations. The data ere collected using GCMS Real Time analysis version 6.1 and the output was visualized using GCMS Post run software version 6.1 (LabSolutions, GCMS Solution; Shimadzu).

### Data analysis

SPSS (Statistical Package for the Social Sciences) was used to perform analyses on the data. Three independent t-tests were carried out on the maximum growth rate of the growth inhibition data, calculated as the change in OD_600 nm_ per hour over the exponential growth phase of the prey. The prey-only control was compared with the negative control, positive control and test compound for each prey species in combination with each metabolite mixture. A Bonferroni-adjusted significance cut-off was used to assess the statistical significance of the effect compared with the prey only at the *P*<0.05 level.

The total ion chromatogram (TIC) from each sample was taken forward for principal component analysis (PCA) using Solo [version 9.0(2022); Eigenvector Research]. The data were first imported into MatLab (MathWorks) using a custom script and then in Solo were pre-processed to be normalized by length, and then mean-centred. Furthermore, generalized least squares (GLS) weighting by strain was applied to declutter the within-group variation to emphasize the between-strain variation. Multiway ANOVA was conducted on the plotted data of the PC scores using SPSS, and a post-hoc least significant difference (LSD) test was applied. The retention times of the largest loadings for each PC were compared to the original TICs to provide putative identifications of these distinguishing metabolites. These compound names were submitted to the online webserver MetaboAnalyst 5.0 (available at: https://www.metaboanalyst.ca/home.xhtml) for biochemical pathway analysis. The raw GC-MS data are available at: DOI 10.17605/OSF.IO/8BZKT. Figs 2 and 4(c) were produced in R version 4.3.1.

## Results

### Strain selection

Evidence suggests that top predators (those which show the highest level of predation in a given timeframe) against a single species are likely to show potent activity against a range of prey. Therefore, strain CA010 [2] was selected as it was previously identified as the best predator against four out of ten different prey species compared with 113 other strains [1]. AB022 [2] and CA010 are grouped differently according to a hierarchical clustering tree based on predation profiles [1], despite the fact that they are members of the same species, so AB022 was also selected for investigation. AB050A^T^ [17] (DSM 108846) is grouped next to AB022 according to predation profile and is the type strain of *Corallococcus aberystwythensis *. Thus far, AB050A^T^ has received little attention but has demonstrated predatory activity against a range of prey, and therefore represents the potential for production of novel compounds [17]. The type strains DSM 14696^T^ and DSM 14675^T^ were selected as separate representatives for the genera *Corallococcus* and *Myxococcus* respectively to allow comparisons across both the species and genus taxonomic levels. According to predation data from Livingstone *et al*. [17], DSM 14696^T^ was ranked as the top predator of ten strains tested [17]. Furthermore, DSM 14675^T^ demonstrated above average predation for all three prey tested by Chambers *et al*. [18]. Six prey species were chosen (Table 1) to represent a diversity of organisms including Gram-positive and Gram-negative bacteria in addition to a fungal species. Predation assays and prey susceptibility were investigated previously regarding these specific prey, which were described as ‘clinically important’, making them relevant prey for the investigation of novel antimicrobial metabolites [1].

### Phylogenetic analysis

**Fig. 1.**
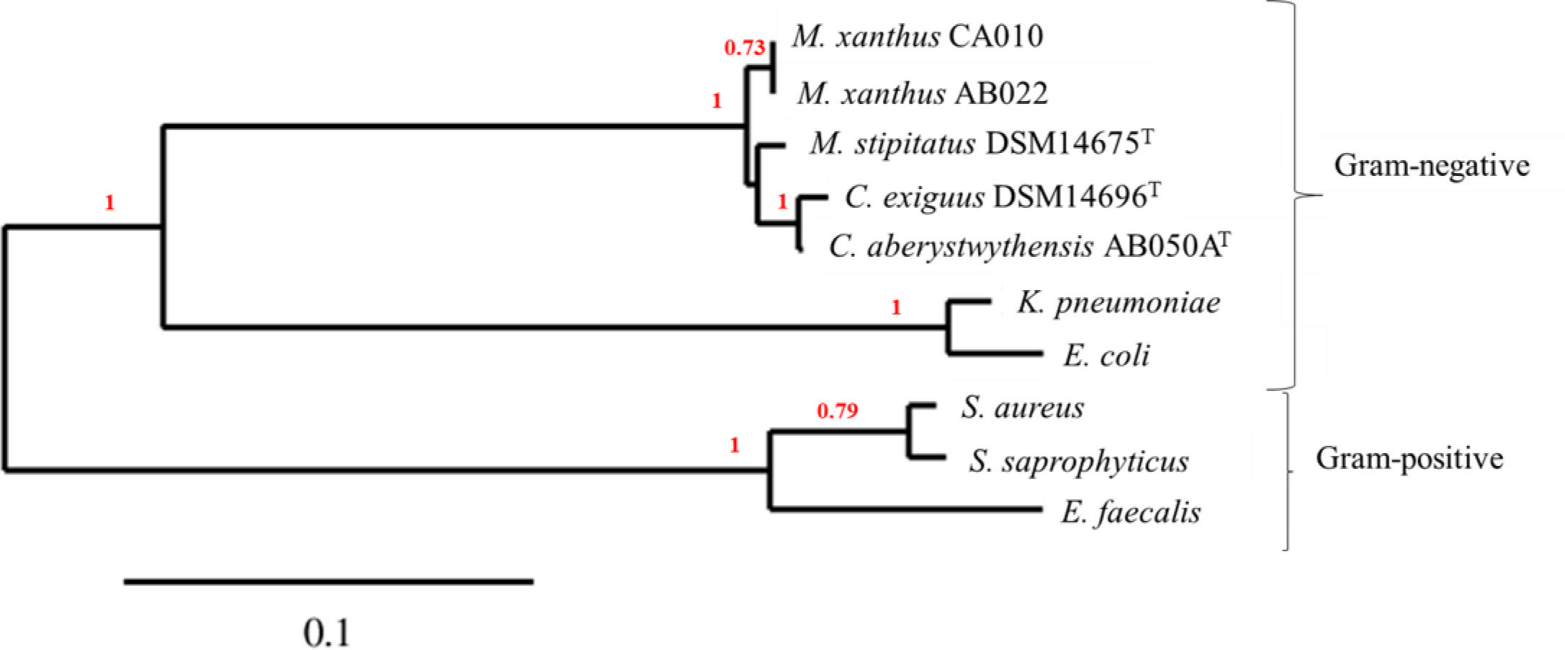
Phylogenetic tree demonstrating the relatedness of bacterial prey and predator species based on 16S rRNA gene sequences. Bootstrap values >0.7 are represented by the red numbers at each node; and the scale bar is representative of the rate of substitution.

To assess the evolutionary relationships between predators and prey, a phylogenetic tree was produced (Fig. 1). High bootstrap numbers give confidence that it is an accurate representation and the groupings in this tree are as would be predicted from current taxonomic classifications. There is obvious delineation between the Gram-positive and Gram-negative prey species and also a clear divide between myxobacteria and prey species. DSM 14696^T^ and AB050A^T^ are both members of the genus *Corallococcus* genus and is clearly shown by their grouping in the tree.

### BGC prediction

To identify potential BGCs in each strain’s genome, antiSMASH analysis was carried out. This revealed that each strain has a different profile of predicted BGCs with differing numbers of regions and types of region(Table 2). The most frequent BGC types (three or more identified across the five strains) represent a mean of 84.5 % (144 out of 170) of all predicted BGCs for each strain. Both *Corallococcus* strains (AB050A^T^ and DSM 14696^T^) contained a higher number of BGCs than the *Myxococcus* strains. The most common types (52 % or 88 out of 170 BGCs) were NRPS (non-ribosomal peptide synthetase) and a combination of NRPS and T1PKS (type 1 polyketide synthase). There were an additional 26 BGC types predicted across the strains which occurred only once or twice. A suggestion for ‘most similar known cluster’ was presented alongside the majority of identified regions to give a putative identification of the metabolite produced by the cluster (see File S1). Only four named clusters were common to all five strains. These were identified as VEPE/AEPE/TG-1 (an NRPS-like region), alkylpyrone-407/alkylpyrone-393 (a T3PKS region), geosmin and carotenoid (both terpene regions). Each of these named clusters was identified once in each of the five strains. However, across all strains there were 78 regions for which there was no suggestion for the metabolite produced, showing there is potential that these regions could code for novel metabolites.

**Table 2.** The numbers and types of the most common BGC regions for each strain as predicted by antiSMASH.

Type of region	AB050A^T^	DSM 14696^T^	DSM 14675^T^	AB022	CA010	Total
NRPS	17	17	5	4	5	48
NRPS/T1PKS	9	9	6	8	8	40
Others	6	7	7	3	3	26
RiPP-like	5	3	4	2	2	16
Terpene	4	4	3	2	2	15
Lanthipeptide class II	3	2	0	1	1	7
RRE-containing	2	1	1	1	1	6
T3PKS	1	1	1	1	1	5
T1PKS	1	1	0	1	1	4
Thioamitides	0	0	1	1	1	3
Total	48	45	28	24	25	170

NRPS, non-ribosomal peptide synthetase; T1PKS, type 1 polyketide synthase; T3PKS, type 3 polyketide synthase; RiPP, ribosomally synthesized and post-translationally modified peptide product; RRE, RiPP recognition element. (NRPS-like and NRPS predictions are combined, as are NRPS-like/T1PKS and NRPS/T1PKS predictions.)

### Growth inhibition assay

**Fig. 2.**
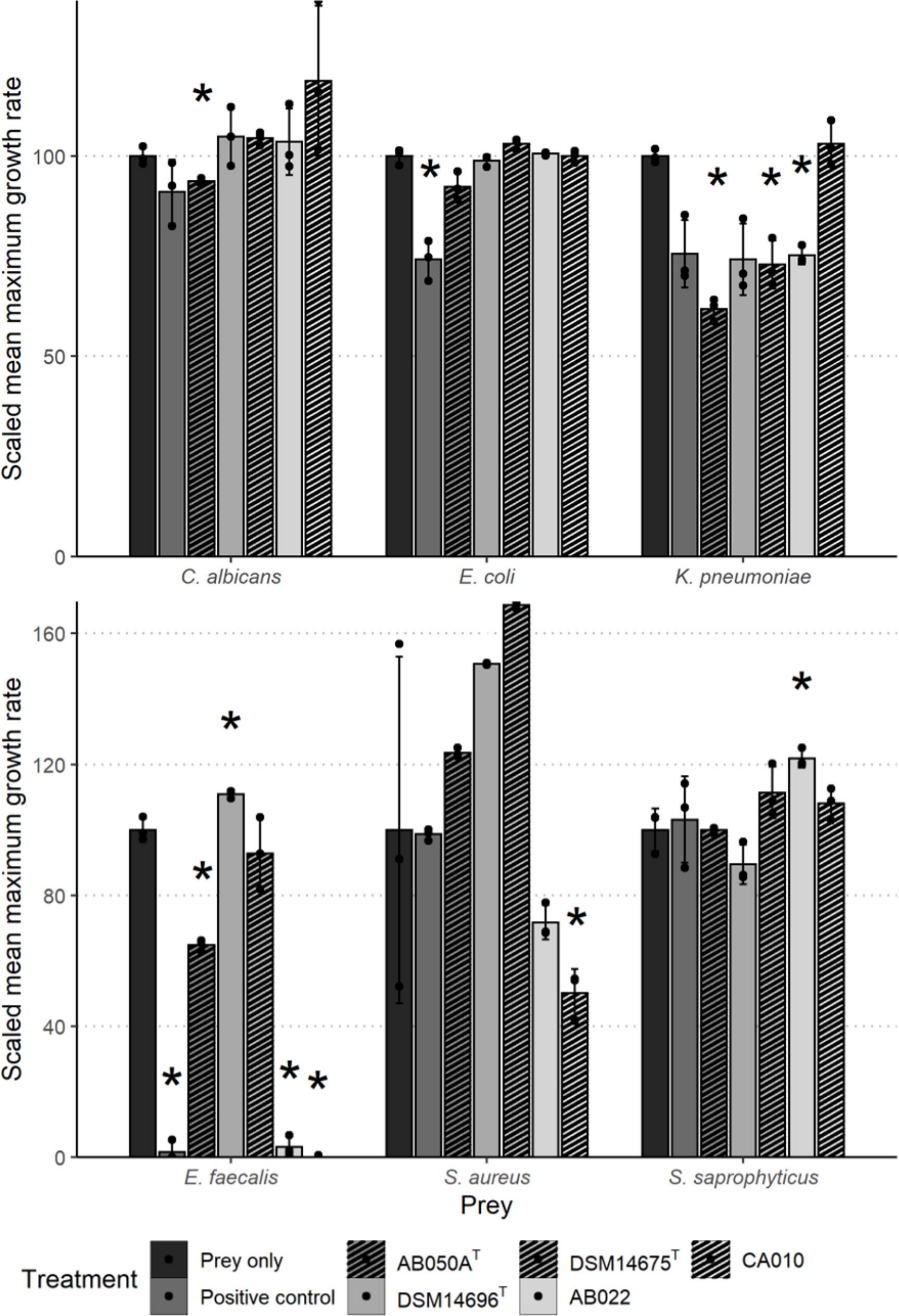
Mean maximum growth rate (change in OD_600nm_ per hour) of prey species (*n*=3) as a percentage of the prey-only control with treatments of metabolite mixtures extracted from the five myxobacterial strains. Positive control consisted of a 1 : 1 mixture of lysozyme (10 mg ml^−1^) and SDS (0.5%). The asterisk (*) denotes a significant difference (*P*<0.05) between the prey-only control and the treatment. Error bars represent ±1 sd from the mean.

A mixture of metabolites for testing was first extracted from triplicate liquid cultures of five myxobacterial predators using Amberlite XAD-16 resin beads. The yield of metabolites from each strain was measured as the mean mass of remaining residue after evaporation of the acetone. The yield was low and similar across all strains with a mean value of 5.2 mg, and a standard deviation of 0.85 mg. Throughout this assay, the OD_600 nm_ of liquid cultures was measured over 24 h to capture the growth and inhibition of growth of the six prey species. The growth of each prey (change in OD_600nm_ per hour) in nutrient media was compared with the growth in combination with the metabolite mixtures. This was repeated for the metabolite mixture from each of the myxobacterial strains. There was no significant reduction in the maximum growth rate when treated with 10 % DMSO as a negative control for any of the prey (although there was a small statistically significant increase (*P*<0.05) with *Escherichia coli *). The positive control resulted in a decrease in the maximum growth rate of all prey species (except *Staphylococcus saprophyticus* which showed increased growth by 3%), with statistically significant decreases (*P*<0.01) seen with *Escherichia coli* and *Enterococcus faecalis* (Fig. 2). Each metabolite mixture demonstrated a different spectrum of activity against the prey (Fig. 2), with statistically significant activity (*P*<0.05) seen by each metabolite mixture against at least one of the prey species. The growth of *Klebsiella pneumoniae* and *Enterococcus faecalis* appeared to most consistently be negatively affected by the metabolite mixtures with significant reduction (*P*<0.05) in growth of *K. pneumoniae* found from three of the five treatments. *Escherichia coli* showed very little change in growth across the treatments with no significant increases or decreases. Surprisingly, the growth of *S. saprophyticus* increased with each treatment, but only significantly so (*P*<0.05) with treatment with metabolite extract from AB022 (an increase of 22%). Similarly, growth of *Candida albicans* increased (although not significantly) with each treatment apart from when treated with the metabolite mixture from AB050A^T^ which resulted in a statistically significant decrease of 7 % (*P*<0.05). The metabolite mixture from AB050A^T^ showed the widest range of antimicrobial activity against the prey (i.e. resulted in decreased maximum growth rate in the greatest number of prey species – 4/6 prey) and that from DSM 14675^T^ showed the smallest (2/6 prey). However, the growth rate of *Enterococcus faecalis* was reduced to zero in combination with CA010, and almost zero with AB022 (highly statistically significant decreases – *P*<0.001). This shows both the potency of the CA010 and AB022 metabolite mixtures, but also the susceptibility of *Enterococcus faecalis. K. pneumoniae* also appears to be more sensitive to the metabolite mixtures than other prey species, with statistically significant decreases in maximum growth rate seen with three of the five metabolite mixtures (*P*<0.01).

### Metabolite characterization

**Fig. 3.**
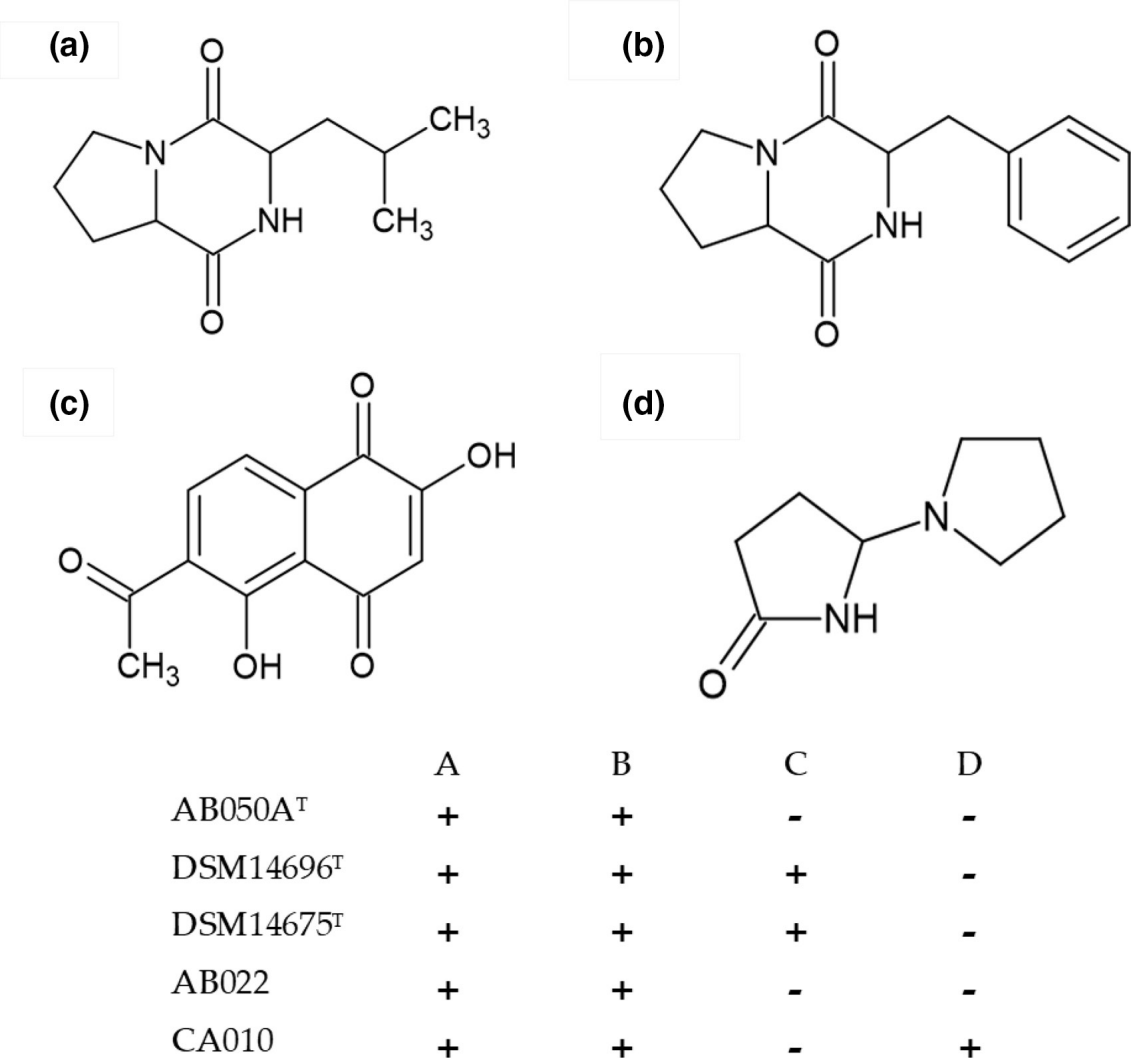
The proposed skeletal structures of the potential compounds A–D identified through GC-MS of the metabolite mixtures and their presence (+) or absence (−) in each mixture as shown in the matrix. A, pyrrolo[1,2a] pyrazine-1,4-dione, hexahydro-3-(2-methyl propyl); B, pyrrolo[1,2a] pyrazine-1,4-dione, hexahydro-3-(phenylmethyl); C, 1,4-naphthoquinone, 6-acetyl-2,5-dihydroxy; D, 5-pyrrolidono-2-pyrrolidone.

GC-MS was used to separate components of the metabolite mixtures and make tentative identifications of compound mass spectra by comparison to known structures in GC-MS libraries (NIST) to provide potential identities for the metabolites. The large dataset was manually curated to remove probable artefacts of derivatization and typical cellular components, and compounds of low abundance. Some of the structures identified are displayed in Fig. 3. It is interesting to note the core structural similarities between compounds A and B which differ only at the 3-position on the pyrazine ring structure with A possessing the 2-methylpropyl group in place of the phenylmethyl moiety found in B. Both A and B were identified in all five strains, whereas D was only identified in CA010. C was common to both DSM 14696^T^ and DSM 14675^T^ but was not identified in any other strain. Many peaks from all strains could not be matched to the library, and so many compounds remain unknown.

### Principal component analysis

**Fig. 4.**
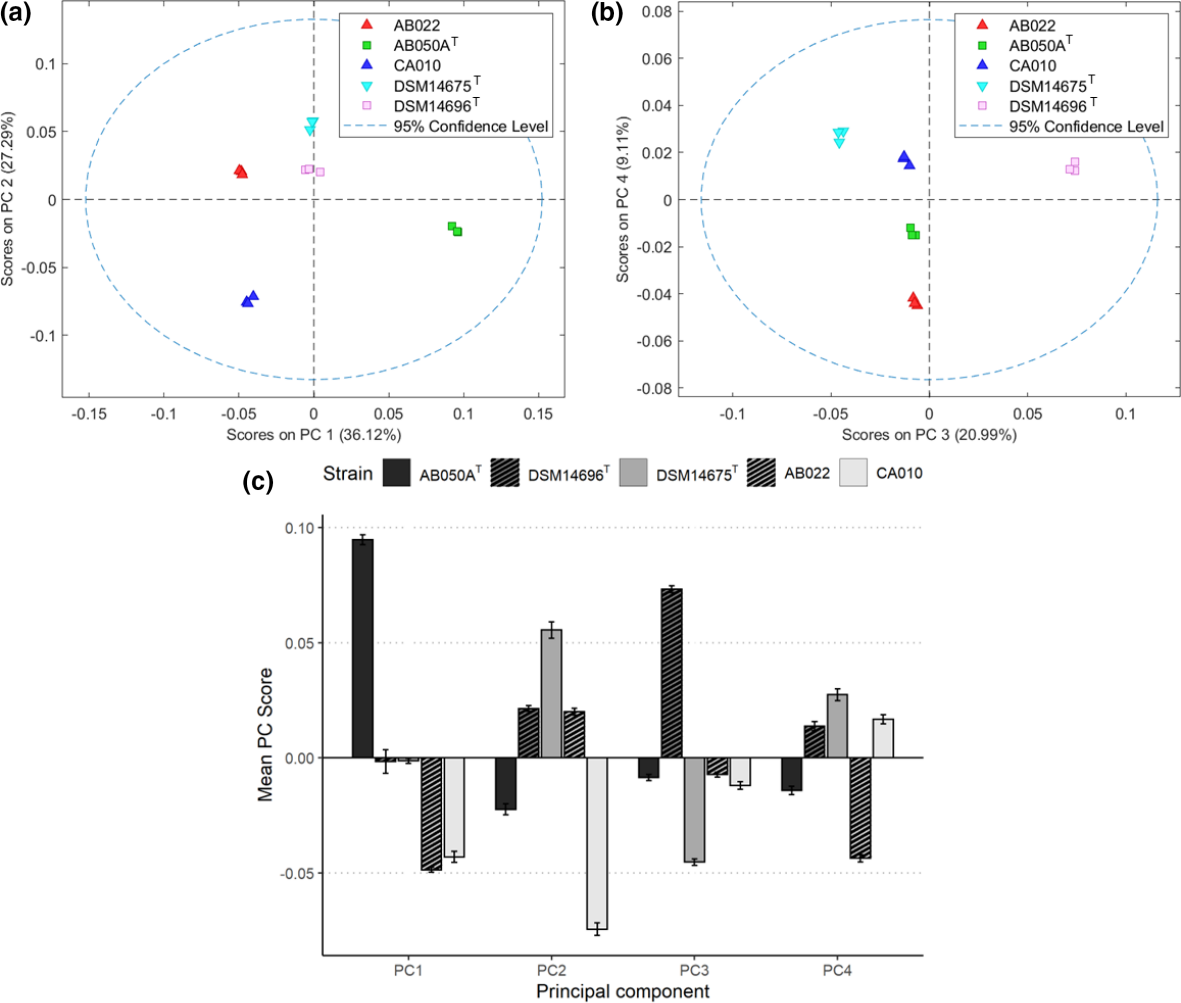
(a, **b**) PCA plots of the TIC for each sample. PC1 against PC2 on the left, and PC3 against PC4 on the right. Each strain is denoted by a different symbol as in the key, all triangles are *Myxococcus* sp. (up are *M. xanthus *, down are *M. stipitatus *) and the squares are *Corallococcus* sp. (**c**) The mean score for each strain within each principal component. Error bars represent ±1 sd from the mean.

Relationships/groupings between the strains based on metabolic profile were assessed by PCA. Cross-validation was achieved by the venetian blinds method, and examination of the root mean square of errors of cross-validation and correlation suggested that a four-component model that explained a total of 93.5 % of the variation in the TIC (File S2) was most suitable. A simpler model that did not employ GLS weighting showed clustering according to strain. The final model in which the decluttering algorithm was used revealed exceptionally strong groups in the data. Scores plots for the model are shown in Fig. 4(a, b). Both *Myxococcus xanthus* strains had similar scores along PC1 and 3, but while AB022 was neutral with regard to PC2, CA010 was negatively scored and vice versa regarding PC4. In contrast, the two *Corallococcus* strains were scored similarly along PC2 and 4 both being neutral, but DSM 14696^T^ was neutral along PC1 and AB050A^T^ was positive, and the inverse was true regarding PC3.

**Fig. 5.**
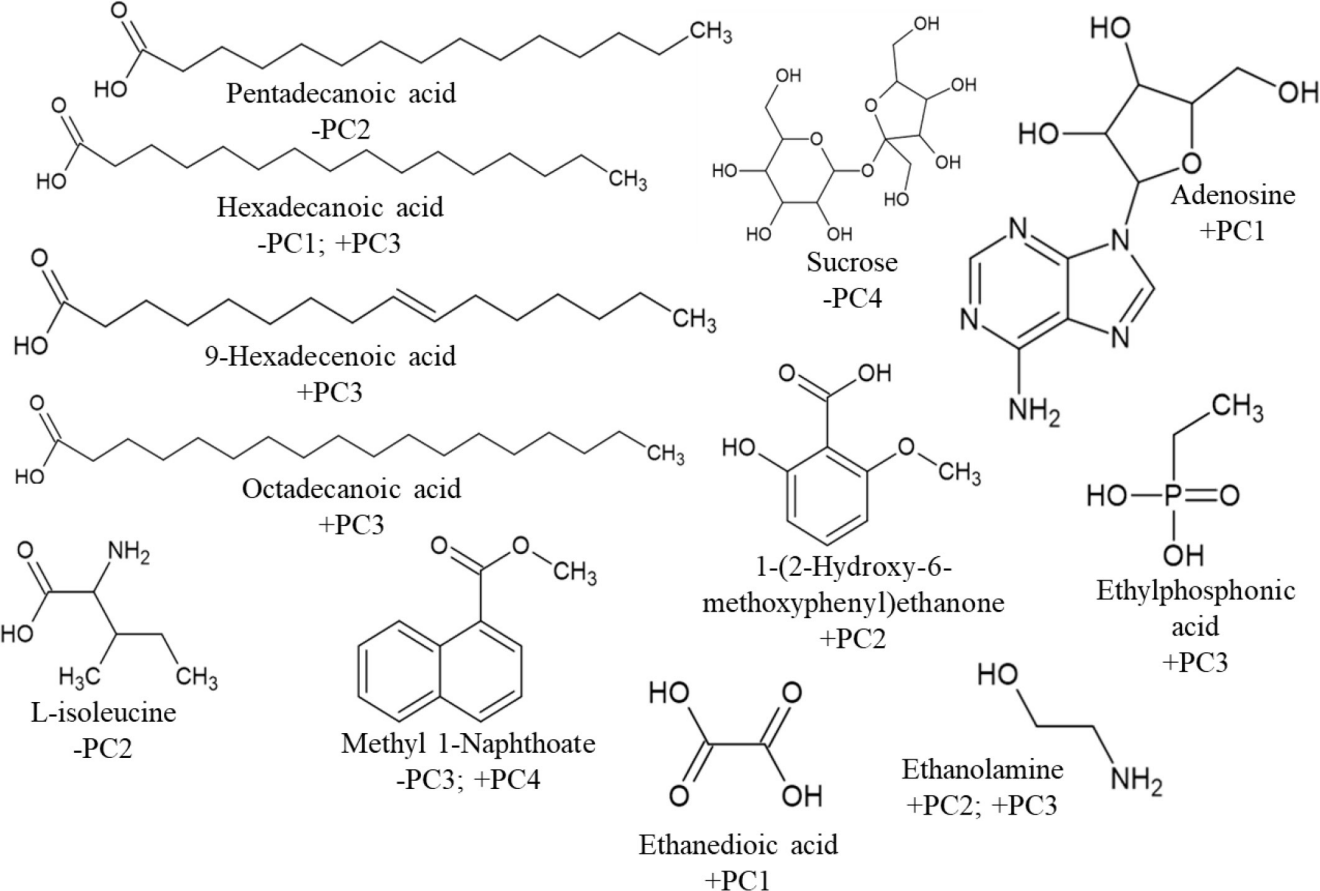
Skeletal structures of metabolites identified as examples of either a positive or negative loading on one or more principal component as labelled below each compound.

Peaks in the sample chromatograms that best matched the retention times of peaks shown in the PC loadings were tentatively identified by library matching. Many compounds were shown to be common for both positive and negative loadings across principal components, but some metabolites were identified that were unique to just one or two PCs (see Fig. 5). Among these discriminating metabolites, at least one unknown compound was present for each PC for which the structures could not be defined. Pathway analysis failed to identify involvement in a common pathway.

## Discussion

Secondary metabolite production plays a key role in predation by myxobacteria, and this study aimed to extract, characterize and assess biological activity of these metabolites from a selection of strains. The five myxobacterial strains were selected for investigation due to being strains with a range of predatory activities (including a top predator) [1], with the view that these strains are more likely to produce antimicrobial metabolites than strains with poor predatory activity. It is known that genomic diversity is high between strains, and that specific genes within the accessory genome are likely to confer prey-specific predatory ability, but the antiSMASH output per strain did not immediately indicate a higher biosynthetic potential for bioactive secondary metabolites for any one of the strains over the others [2]. During extraction of metabolite mixtures, a similar yield of mass of crude extract per volume was obtained of 0.052 g l^−1^ which is at the low end of the range (0.05–0.19 g l^−1^) as reported previously for myxobacteria [7, 19]. This demonstrates that the method used here has comparable efficiency to others previously described.

To test the lytic capability of the metabolite mixtures as a measure of potency, a seeded agar assay adapted from Li *et al*. was used [20]. In that study, the authors were able to successfully demonstrate lysis of the embedded prey due to the addition of their test compounds (outer membrane vesicles - OMVs), which relied on the prey being alive but not being able to actively grow and reproduce and repair cell wall damage. In the current study no visible lysis zones could be produced by addition of the test compounds, suggesting no lytic activity. In addition, viable prey was recovered from agar samples taken from all spots treated with the test compounds. Although this suggests there is no permanent bacteriostatic effect from the test compounds, the potential of a bacteriostatic mode of action cannot be excluded. Therefore, a growth inhibition assay was carried out, in which each metabolite mixture was tested at a concentration of 0.04 g ml^−1^. A spectrum of activity against a range of prey was recorded. Prey species include two of the six ‘ESKAPE’ pathogens (* Staphylococcus aureus* and *Klebsiella pneumoniae *) and *Enterococcus faecalis* (a very close relative to the ESKAPE pathogen *Enterococcus faecium *) which are of key focus due to their increasing prevalence in hospital infections and increasing resistance to antibiotics [21]. Therefore, it is crucial that novel treatments for combating these pathogens are investigated [22]. No significant growth inhibition was seen against *Escherichia coli *, but three of the five mixtures showed significant activity against *K. pneumoniae *. Three different mixtures showed significant activity against *Enterococcus faecalis,* and the mixture from AB050A^T^ significantly inhibited growth of *Candida albicans*. This is interesting as it reflects the diversity of the myxobacterial prey range, including Gram-positive and Gram-negative bacteria as well as fungi. Predation assays from previous studies have given evidence that all five strains assessed here can prey upon Gram-positive and Gram-negative bacteria as well as fungi [1, 17, 18]. Thus, the lack of activity against *Escherichia coli* from any of the myxobacterial metabolite mixtures is surprising, especially considering the widespread use of this species as a typical Gram-negative prey in many assays and applications. It was reported in previous studies that DSM 14675^T^ displayed above average predation and CA010 was the best predator against *Escherichia coli* [1, 18], yet in the growth inhibition assay described here there was very little absolute change in the treated prey cell density compared to the control suggesting the compounds are not effective against *Escherichia coli *. Furthermore, the same study suggested DSM 14675^T^ is also an above average predator against the fungal species *Ustilago maydis* [18], but it had no effect on the fungus *Candida albicans* used here. A further study showed the predatory activity of DSM 14696^T^ to be much greater than that of AB050A^T^ and so it was expected that this might also be reflected in metabolite activity [17]. However, the inhibitory activity of the DSM 14696^T^ metabolite mixture was poorer than that of AB050A^T^ in this study, suggesting that there are other factors aside from metabolite activity that result in an enhanced predatory phenotype, such as protein toxins or digestive enzymes.

In contrast to the expected results, following treatment with four of the five mixtures, *S. saprophyticus* showed increased growth, and significantly so (*P*<0.01) following treatment with the AB022 mixture. It is possibile that this species is less susceptible to attack by predators than other prey as in a hierarchical clustering model it was positioned in a clade adjacent to *P. aeruginosa* which is recognized as being particularly impervious to predation [1]. Furthermore, in that same model *S. aureus* was grouped in the same clade as *S. saprophyticus* and it too showed increased (although not significantly so) growth in combination with three of the five mixtures. Therefore, perhaps the metabolite dose was not sufficiently high to have an adverse effect against this prey. Alternatively, *S. saprophyticus* may be metabolizing the myxobacterial metabolite mixtures more efficiently than the nutrients from the complex media, resulting in enhanced growth.

Following extraction of the metabolite mixtures from the five myxobacterial strains, a preliminary protocol run in singlicate revealed the four compounds shown in Fig. 3 as being of interest, but repeats in triplicate which were required to generate the data for the PCA analysis did not identify compounds C or D in any metabolite mixture. It is likely that the concentration of these compounds was close to the detection limit. Both compounds A and B were identified from all five metabolite mixtures, suggesting they may be core myxobacterial metabolites. The high degree of similarity between the two may suggest they are intermediates within the same biochemical pathway or made by the same enzyme. Compound A has previously been synthesized and shown to have antimicrobial activity [23]. The peptide bond in compound A plays a key role in its antimicrobial properties [23]. The presence of the di-keto character resulting in two peptide bonds in both A and B [also known as cyclo(pro-leu) and cyclo(phe-pro) respectively] produces increased activity compared to a single peptide bond by a factor of over 60 [23]. Both A and B contain a diketopiperazine (DKP) core, these are produced by a variety of microorganisms, and have diverse structures and activities [24]. The ring structure is important in providing rigidity and stability against proteolysis which would impact the bioactivity of the compound, thus making the DKP base an ideal candidate for drug development as it is biologically active, but also stable [24].

Compound A has antimicrobial activity against Gram-positive and Gram-negative prey bacteria and was more effective than a 2-ketopiperazine analogue (one peptide bond compared to the two in compound A) against all bacteria tested apart from *Staphylococcus epidermis* against which they showed equal activity measured by MIC [23]. It showed greatest activity against *K. pneumoniae* and second greatest against *Escherichia coli *. However, the same study demonstrated that although A displays low MIC values in the range 8–64 μg ml^−1^ depending on the prey, it was far less potent than the antibiotic ciprofloxacin (MIC=0.5–2 μg ml^−1^). Thus, further optimization of the compound may yield improved potency. This study reports that *K. pneumoniae* was one of the most affected prey species by the metabolite mixtures, and since A also shows particular activity against this prey a putative link can be suggested that the presence of A in each metabolite mixture is contributing to the bioactivity of the metabolite mixtures. However, a different DKP compound named Sorazinone A and its structural relative (not a DKP) Sorazinone B, which were extracted from the myxobacterium *Sorangium cellulosum* Soce895, did not exhibit strong antimicrobial activity despite the DKP core, suggesting the structure of the adjacent groups to the DKP core also play a key role in its potency [25]. The formation of DKPs is catalysed by enzymes in the NRPS and the CDPS (cyclodipeptide synthase) families [24]. From the BGC analysis, no CDPSs were identified in the myxobacteria strains investigated here. However, NRPSs were one of the most commonly identified BGCs among all strains, and therefore could potentially produce compounds A and B.

The PCA model produced from the TIC of each sample allows the groupings within the data to be visualized. It is important to remember that whilst this model employed generalized least squares weighting by strain that helped to declutter ‘noise’ from the ‘signal’ and so enhance grouping in the scores plots, it can only do this if groupings are already present. It does not falsify grouping of the data according to its labels, but rather it weights the data according to its group and therefore produces greater definition between groupings. It is interesting that the 16S-based phylogeny of the strains is not reflected in the groupings based on metabolite profile but given that a metabolite profile can be predicted by its genome, and this is highly variable even within a single species, it is perhaps unsurprising [26]. Furthermore, it has already been reported that prey range is not dependent on phylogeny and since secondary metabolite production has a key function in predatory activity which dictates prey range, the evidence from this investigation further supports this finding [1].

Looking at the loadings of each component in the PCA model, specific retention times of the TIC which represent peaks for certain metabolites can be identified, which explain the groupings presented in the model. A variety of metabolites were putatively identified by comparing mass spectra to GC-MS libraries. With each identification a percentage certainty was provided. The mean certainty was 81 %, suggesting it is highly likely that the majority of compounds have been correctly identified. The highest certainty was 95 % for the identification of compound A, and the lowest certainty was 63 %. Furthermore, across the triplicate repeats for each strain, identifications were consistent providing further confidence in the identifications. Nonetheless, as the identifications provided are based on database matches and not direct predictions from the mass spectra, there is a chance that identifications are provided that although similar are ultimately different from the true structure. Furthermore, any metabolites that cannot be matched to the libraries will probably remain as unknown. There were many peaks across the TICs for all strains for which the metabolite structure could not be identified. Therefore, this represents the potential for novel metabolite discovery. With the vast genomic diversity among myxobacteria and the ever-expanding accessory genome, with every new genome added to consideration, there is substantial potential for genes encoding novel metabolites that await discovery. To investigate these unknowns further, additional analysis of the samples through alternative MS methods, for example direct infusion LC–MS [27], could help to provide a chemical formula for each metabolite which can then be used to provide predictions of its structure.

Compound A is of particular interest due to its antimicrobial properties, and it was identified as having a large loading on all principal components in a positive direction on PC1, 3 and 4, and negatively on PC2. However, it is clear that the presence of this compound alone cannot separate the strains as it is a combination of metabolites with positive and negative loadings that produce the specific score for each strain, and it is difficult to extract the specific effect of compound A. Unfortunately, when the list of compounds with the greatest loadings was submitted to MetaboAnalyst, there was no hit for the chemical name of compound A so at this stage there is no further evidence as to the biochemical pathways it is involved in. However, many of the compounds did have matches, and they are mainly involved in production and degradation of fatty acids, amino acids and glycerophospholipids. This is not unexpected as these indicate normal growth and function.

Many compounds including secondary metabolites are actively secreted in OMVs [28]. Strains within a species have similarities in their OMV proteome, but also a high amount of variation [29]. It is likely that OMVs from different strains also possess a variety of secondary metabolites as well as proteins, giving them varying antimicrobial activities [30]. OMVs have been shown to cause prey lysis and growth inhibition on solid media, and this activity appears to be caused by the OMV contents and not the vesicles per se [20, 31]. Therefore, it is suggested that OMVs may be important as transport vessels for bioactive cargo and may represent a higher yielding source of bioactive metabolites than extraction from cells. Furthermore, assessment of the metabolites produced in different growth media, with co-incubation with prey and from a wider range of myxobacterial strains, could give valuable insights into how different factors can alter metabolite profiles for optimization and potential development.

## Conclusions

The study presented here demonstrates the extraction, characterization and antimicrobial activity of metabolite mixtures secreted by five myxobacterial strains. It is shown that although the traditional method of extraction using XAD-16 resin beads is successful, it is not especially efficient and alternative sources of metabolites including OMVs should be considered. Of the metabolites extracted and putatively elucidated using GC-MS, many represent normal metabolic function including synthesis and degradation of fatty acids and amino acids. However, compound A (pyrrolo[1,2a] pyrazine-1,4-dione, hexahydro-3-(2-methyl propyl)) and its analogue compound B (pyrrolo[1,2a] pyrazine-1,4-dione, hexahydro-3-(phenylmethyl)) contain a DKP core which has previously been shown to have antimicrobial properties. The metabolite mixture from each of the five strains showed significant (*P*<0.05) activity against at least one of the six prey strains tested. *Enterococcus faecalis* and *K. pneumoniae* appear to be most negatively affected by the myxobacterial metabolites and surprisingly there was very little effect on *Escherichia coli *. PCA revealed that each strain possesses a unique profile of metabolites which does not appear to correlate with their 16S-based phylogeny. This further supports the notion that secondary metabolites (alongside other factors) play an important role in predation and that predatory activity in combination with prey range are characters independent of 16S-based phylogeny. Therefore, the relatedness of strains (based on their 16S identity) is not a good predictor of secondary metabolism or predatory potential. We hypothesize that compound A identified here within the metabolite mixtures contributes to the inhibition of prey growth. However, many compounds from the mixtures were unable to be matched in GC-MS libraries and their structure remains unknown. It is likely that among these novel metabolites are core structures which may also contribute to the antimicrobial activity.

## Supplementary Data

Supplementary material 1

Supplementary material 2
